# Association between the *VEGFR-2* -604T/C polymorphism (rs2071559) and type 2 diabetic retinopathy

**DOI:** 10.1515/biol-2022-0081

**Published:** 2023-03-03

**Authors:** Yazhen Yuan, Chenjun Shao, Yongqing Guan, Hongwei Lu, Dandan Wang, Shuangmei Zhang

**Affiliations:** Department of Ophthalmology, The Fourth Hospital of Hebei Medical University, No. 12, Jian Kang Road, Shijiazhuang, 050019 Hebei, China

**Keywords:** type 2 diabetes, diabetic retinopathy, vascular endothelial growth factor receptor, gene polymorphism, *VEGFR-2*

## Abstract

This retrospective case–control study examined the association between the rs2071559 (-604T/C) single nucleotide polymorphism (SNP) in the *vascular endothelial growth factor receptor* (*VEGFR*)*-2* gene and the risk of diabetic retinopathy (DR) in Northern Han Chinese. This study included patients diagnosed with diabetes mellitus (DM) in Shijiazhuang between 07/2014 and 07/2016. The healthy controls were unrelated individuals who received routine physical examinations. The diabetic patients were grouped as DM (diabetes but no fundus examination abnormalities), proliferative DR (PDR), and non-proliferative DR (NPDR). Finally, 438 patients were included: 114 controls and 123, 105, and 96 patients in the DM, NPDR, and PDR groups, respectively. In the multivariable analyses and all genetic models, the *VEGFR-2* rs2071559 SNP was not associated with DR (among all diabetic patients) or with PDR (among the patients with DR) after adjustment for age, sex, duration of DM, blood glucose, systolic blood pressure, diastolic blood pressure, and body mass index (all *P* > 0.05). In conclusion, the *VEGFR-2*- 604T/C rs2071559 SNP is not associated with DR or PDR in the Han Chinese population of Shijiazhuang (China).

## 1 Introduction

Diabetic retinopathy (DR) is a microvascular complication of type 2 diabetes mellitus (T2DM) and a leading cause of preventable blindness in adults [1–3]. DR is a spectrum that starts with non-proliferative DR (NPDR) that eventually progresses to proliferative DR (PDR) [1–3]. The worldwide prevalence of DR is 24.6% in patients with diabetes [4], the highest incidence rates are observed in African Americans, Hispanics, and South Asians [3]. In China, among patients with diabetes, the prevalence rates of DR and vision-threatening DR are 27.9 and 12.6%, respectively [5]. The management of DR includes glycemic control, lipidemia control, blood pressure control, laser therapy, intravitreal anti-vascular endothelial growth factor (VEGF) therapy, vitrectomy, and intravitreal steroids [2,6].

VEGF is a key molecule in the development of DR [7,8]. Intravitreal VEGF levels are high in diabetic patients and correlate with neovascularization [9], especially in patients with PDR [3,10]. In addition to VEGF levels, the expression and distribution of the VEGF receptor (VEGFR)-2 are also crucial in developing DR and progressing from NPDR to PDR [11–13]. Indeed, VEGFR-2 controls endothelial cell proliferation, migration, survival, and osmosis via its activation by VEGF [8].

Single nucleotide polymorphisms (SNPs) in the *VEGFR-2* gene can affect the development and progression of multiple diseases by affecting the binding efficiency between VEGFR-2 with VEGF or by reducing the transcriptional activity of VEGFR-2 [14–16]. The involvement of some *VEGF* SNPs in the development of DR and response to treatment are well-known [17–20], but the role of *VEGFR-2* SNPs in the development of DR is poorly known. The rs2071559 SNP is located in the promoter of *VEGFR-2* and decreases the transcriptional activity of VEGFR-2 [14,21,22]. A previous study revealed that four SNPs in the *VEGF* gene and one (rs2071559 or -604T/C) in the *VEGFR-2* gene were associated with the development of DR [23], but it did not examine the association of the rs2071559 SNP with PDR. There are several studies on the polymorphisms of *VEGF* in DR [17,23–25], but only one study of the rs2071559 SNP in *VEGFR-2* was found [23]. Since DR is a complex trait affected by multiple genes and the environment, exploring the role of *VEGFR-2* SNPs in various populations is relevant.

Therefore, the present study aimed to examine the association between the -604T/C locus polymorphism of the *VEGFR-2* gene and the risk of DR among T2DM patients and then of PDR among DR patients in a Han population in North China.

## 2 Materials and methods

### 2.1 Study design and subjects

This retrospective case–control study included patients with T2DM who visited the Department of Ophthalmology and the Department of Endocrinology of the Fourth Hospital of Hebei Medical University between July 2014 and July 2016. T2DM was diagnosed according to the International World Health Organization (WHO) T2DM diagnostic criteria (1999) [26]. The healthy controls were unrelated individuals who received routine physical examinations between July 2014 and July 2016. All subjects were of Han ethnicity and from families living in Shijiazhuang, Hebei Province, China, for at least three generations.

The subjects were divided into four groups (one control group and three patient groups) according to the 2002 International Clinical Classification Criteria for DR [27]. The DM group included the T2DM patients without fundus examination abnormalities. The PDR group included the patients with one or more of the following: neovascularization, vitreous hemorrhage, and preretinal hemorrhage. The NPDR group included the patients with microangioma, intraretinal hemorrhage, and microvascular abnormalities, but without the PDR-specific findings.


**Informed consent:** Informed consent has been obtained from all individuals included in this study.
**Ethical approval:** The research related to human use has been complied with all the relevant national regulations and institutional policies and in accordance with the tenets of the Helsinki Declaration, and has been approved by the ethics committee of The Fourth Hospital of Hebei Medical University.

### 2.2 Data collection

The data were obtained from the medical records and included sex, age, fasting blood glucose levels, body height, body weight, systolic blood pressure, diastolic blood pressure, and T2DM duration. The body mass index (BMI) was calculated as BMI = body weight (kg)/height (m^2^).

### 2.3 *VEGFR-2* -604T/C polymorphism

DNA was routinely extracted from venous blood collected in sodium citrate tubes and stored at 4°C. Peripheral blood leukocyte DNA was extracted using the proteinase K digestion-saturated sodium chloride salting-out method within 1 week of blood collection [28].

The polymerase chain reaction-ligation detection reaction (PCR-LDR) method was used to determine the *VEGFR-2* -604T/C polymorphism. The forward primer for the amplification of rs2071559 was 5′-AAA TAT TTT GGG AAA TAG CGG GAA TG-3′, and the reverse primer was 5′-TGG CGA ACT GGG CAA GTG CGT TTT C-3′. The forward and reverse primers for -604T/C were 5′-GAG CAC GAT GGA CAA AAG CCT-3′ and 5′-GGG AGA AGC GGA TAC TCA GCC-3′, respectively. The PCR product length was 255 bp, and the GC content was 47.5%. The LDR probe design included two probes. The sequences of the probes for rs2071559 were TC: 5′-TTT TTT TTA TAT TTT GGG AAA TAG CGG GAA TGC-3′, TT: 5′-TTT TTT TTT TTA TAT TTT GGG AAA TAG CGG GAA TGT-3′, and TR: 5′-P-TGG CGA ACT GGG CAA GTG CGT TTT CTT TTT T-FAM-3′.

The size of the ligated products was 64/C and 67/T. The LDR reaction consisted of cycling through the following conditions for 25 cycles: 94°C for 30 s and 56°C for 1 min.

Sterile distilled water was used as a negative control. In addition, 10% of the DNA samples were randomly selected for duplicate experiments. All repeated results were consistent with the original ones.

### 2.4 Statistical analysis

Statistical analysis was performed using SPSS 13.0 (SPSS, Chicago, IL, USA). The Hardy–Weinberg equilibrium was tested in all groups using the chi-square test. Normally distributed continuous variables were expressed as means ± standard deviations (and analyzed using one-way analysis of variance and the Bonferroni *post hoc* test. Non-normally distributed continuous variables were presented as medians (ranges) and compared using the Kruskal–Wallis test. Non-conditional logistic regression analysis was performed to calculate the odds ratios (ORs) and 95% confidence intervals (95% CIs). *P*-values < 0.05 were considered statistically significant.

## 3 Results

### 3.1 Characteristics of the subjects

Finally, 438 subjects were included: 114 controls, 123 patients in the DM group, 105 in the NPDR group, and 96 in the PDR group. There were no significant differences in age and sex among the four groups (all *P* > 0.05) (Table A1).

### 3.2 Association between *VEGFR-2* gene rs2071559 and DR

The three genotypes (CC, CT, and TT) were present in all four groups and met the Hardy–Weinberg equilibrium (all *P* > 0.05) (Table A2). The frequencies of CC, CT, and TT were 17.6, 45.6, and 36.8% in the control group, 12.2, 50.4, and 37.4% in the DM group, 7.6, 53.3, and 39.1% in the NPDR group, and 7.3, 46.9, and 45.8% in the PDR group. There were no significant differences among the four groups (*P* = 0.191) (Table 1).

**Table 1:** Distribution of genotype and allele frequencies of *VEGFR*-2 -604T/C

	Control group *N* = 114	DM group *N* = 123	NPDR group *N* = 105	PDR group *N* = 96	*P*
Genotype, *n* (%)					0.191
CC	20 (17.6%)	15 (12.2%)	8 (7.6%)	7 (7.3%)	
CT	52 (45.6%)	62 (50.4%)	56 (53.3%)	45 (46.9%)	
TT	42 (36.8%)	46 (37.4%)	41 (39.1%)	44 (45.8%)	
Allele, *n* (%)					0.198
C	92 (40.4%)	92 (37.4%)	72 (34.3%)	59 (30.7%)	
T	136 (59.6%)	154 (62.6%)	138 (65.7%)	133 (69.3%)	

DM, diabetes mellitus without DR group; NPDR, non-proliferative DR group; PDR, PDR group.

The C and T allele frequencies were 40.4 and 59.6% in the control group, 37.4 and 62.6% in the DM group, 34.3 and 65.7% in the NPDR group, and 30.7 and 69.3% in the PDR group (*P* = 0.198) (Table 1).

### 3.3 Univariable and multivariable analyses in the T2DM patients

The rs2071559 genotypes were not associated with DR in the univariable analysis (*P* > 0.05) (Table 2). The duration of T2DM (OR = 1.064, 95% CI: 1.024–1.105, *P* = 0.002), blood glucose (OR = 1.801, 95% CI: 1.057–3.067, *P* = 0.030), systolic blood pressure (OR = 1.023, 95% CI: 1.009–1.038, *P* = 0.001), and diastolic blood pressure (OR = 1.047, 95% CI: 1.021–1.075, *P* < 0.001) were associated with DR. The rs2071559 SNP of the *VEGFR-2* gene was not associated with DR after adjustment for age, sex, duration of DM, blood glucose, systolic blood pressure, diastolic blood pressure, and BMI (Table 3).

**Table 2:** Univariable logistic regression analysis for factors associated with DR among type 2 diabetic patients

		OR	95% CI	*P*
**Risk factor**				
Age	>60 vs ≤60	1.008	(0.643, 1.58)	0.971
Sex	Female vs male	1.073	(0.684, 1.685)	0.759
Duration of DM	Per one year	1.064	(1.024, 1.105)	0.002
Blood glucose	>6.1 vs ≤6.1 mmol/L	1.801	(1.057, 3.067)	0.030
SBP	Per one mmHg	1.023	(1.009, 1.038)	0.001
DBP	Per one mmHg	1.047	(1.021, 1.075)	<0.001
BMI	18.5–24	Ref.	—	—
	≤18.5	1.063	(0.187, 6.047)	0.945
	>24 vs 18.5-24	0.794	(0.496, 1.271)	0.337
**Genotype**				
Dominant model	(TC + CC) vs TT	0.815	(0.515, 1.292)	0.384
Recessive model	(TC + TT) vs CC	0.581	(0.273, 1.234)	0.158
Co-dominant model	TC vs TT	0.541	(0.243, 1.205)	0.133
	CC vs TT	0.882	(0.546, 1.422)	0.606
Over-dominant model	(TT + TC) vs TC	1.006	(0.642, 1.576)	0.978

DM, diabetes mellitus; SBP, systolic blood pressure; DBP, diastolic blood pressure; BMI, body weight index; OR, odds ratio; CI, confidence interval.

**Table 3:** Multivariable logistic regression analysis of the factors associated with DR among type 2 diabetic patients using different genetic models

Genetic models	Genotype	Adjusted OR	95% CI	*P*
Dominant model	TT	Ref.		
	TC/CC	0.929	(0.567, 1.521)	0.769
Recessive model	TT/TC	Ref.		
	CC	0.495	(0.218, 1.121)	0.092
Co-dominant model	TT	Ref.		
	CC	0.506	(0.213, 1.204)	0.123
	TC	1.041	(0.623, 1.74)	0.877
Over-dominant model	TC	Ref.		
	TT/CC	0.841	(0.519, 1.363)	0.483

Adjusted for age, sex, duration of diabetes mellitus, blood glucose, SBP, DBP, and BMI.

OR, odds ratio; CI, confidence interval.

### 3.4 Univariable and multivariable analyses among patients with DR

The rs2071559 genotypes were not associated with PDR in the univariable analysis (*P* > 0.05) (Table 4). Female sex (OR = 1.776, 95% CI: 1.010–3.125, *P* = 0.046), duration of T2DM (OR = 1.082, 95% CI: 1.032–1.134, *P* = 0.001), systolic blood pressure (OR = 1.018, 95% CI: 1.002–1.034, *P* = 0.032), and BMI > 24.0 kg/m^2^ (OR = 0.358, 95% CI: 0.198–0.645, *P* = 0.001) were associated with the development of PDR. The rs2071559 SNP of the *VEGFR-2* gene was not associated with PDR in any genetic model after adjustment for age, sex, duration of DM, blood glucose, systolic blood pressure, diastolic blood pressure, and BMI (Table 5).

**Table 4:** Univariable logistic regression analysis for factors associated with PDR among DR patients

		OR	95% CI	*P*
**Risk factor**				
Age	>60 vs ≤60	0.842	(0.484, 1.466)	0.544
Sex	Female vs male	1.776	(1.01, 3.125)	0.046
Duration of DM	Per one year	1.082	(1.032, 1.134)	0.001
Blood glucose	>6.1 vs ≤6.1 mmol/L	1.552	(0.743, 3.244)	0.242
SBP	Per one mmHg	1.018	(1.002, 1.034)	0.032
DBP	Per one mmHg	1.013	(0.986, 1.041)	0.349
BMI	18.5–24	Ref.	—	—
	≤18.5	0.193	(0.019, 1.946)	0.163
	>24 vs 18.5–24	0.358	(0.198, 0.645)	0.001
**Genotype**				
Dominant model	(TC + CC) vs TT	0.757	(0.432, 1.327)	0.331
Recessive model	(TC + TT) vs CC	0.954	(0.332, 2.737)	0.930
Co-dominant model	TC vs TT	0.815	(0.271, 2.449)	0.716
	CC vs TT	0.749	(0.42, 1.336)	0.327
Over-dominant model	(TT + TC) vs TC	1.295	(0.744, 2.256)	0.361

PDR, proliferative DR; DM, diabetes mellitus; SBP, systolic blood pressure; DBP, diastolic blood pressure; BMI, body weight index; OR, odds ratio; CI, confidence interval.

**Table 5:** Multivariable logistic regression analysis of the factors associated with PDR among DR patients in different genetic models

Genetic models	Genotype	Adjusted OR	95% CI	*P*
Dominant model	TT	Ref.		
	TC/CC	0.868	(0.456, 1.651)	0.665
Recessive model	TT/TC	Ref.		
	CC	1.207	(0.355, 4.102)	0.763
Co-dominant model	TT	Ref.		
	CC	1.092	(0.303, 3.931)	0.893
	TC	0.842	(0.435, 1.629)	0.609
Over-dominant	TC	Ref.		
	TT/CC	1.205	(0.641, 2.262)	0.563

Adjusted for age, sex, duration of diabetes mellitus, blood glucose, SBP, DBP, and BMI.

PDR, PDR; OR, odds ratio; CI, confidence interval.

## 4 Discussion

While the role of *VEGF* SNPs in the development of DR and response to treatment has been described [18,20], the role of *VEGFR-2* SNPs in the development of DR is poorly known. Therefore, this study aimed to examine the association between the rs2071559 SNP of the *VEGFR-2* gene and the risk of DR in a Han population in North China. The results suggest that the *VEGFR-2* -604T/C polymorphism (rs2071559) is not associated with DR among T2DM patients or PDR among patients with DR in the Han population of Shijiazhuang (China).

DR is a complex trait affected by numerous genes and environmental factors [29,30], and the pathogenesis is still unclear. Of note, DR is a polygenic trait affected by numerous genes, and any polymorphisms within each of these genes will influence the development of DR [29,30]. VEGF is a key molecule in the development of DR [7,8]. Intravitreal VEGF levels are high in diabetic patients and correlate with neovascularization [9], especially in patients with PDR [3,10]. In addition to VEGF levels, the expression and distribution of the VEGF receptor (VEGFR)-2 are also important in developing DR and progressing from NPDR to PDR [11–13]. Indeed, VEGFR-2 controls endothelial cell proliferation, migration, survival, and osmosis via its activation by VEGF [8]. Still, other receptors are involved in DR. VEGFR belongs to the receptor tyrosine kinase superfamily and is a membrane mosaic protein. It contains three subtypes: VEGFR-1 (Flt-1), VEGFR-2 (KDR/Flk-1), and VEGFR-3 (Flt-4) [31,32]. In addition, there are some related receptors, such as neuropilin 1 and 2 [33]. Among them, VEGFR-1 is mainly expressed in monocytes, trophoblast cells, renal mesangial cells, and vascular endothelial cells [31,32]. It is known that VEGF-A, VEGF-B, and PIGF can be combined with it, which is mainly related to the growth regulation of hematopoietic stem cells. VEGFR-2 is mainly distributed on the surface of endothelial cells [31,32]. Other cells include hematopoietic stem cells, retinal precursor cells, and megakaryocytes, which bind to VEGF-A, VEGF-C, VEGF-D, and VEGF-E [31,32]. Combined with VEGF, it stimulates endothelial cell proliferation, increases vascular permeability, and promotes angiogenesis [31,32]. VEGFR-3 is mainly expressed in lymphatic endothelial cells [31,32]. It is known that VEGF-C and VEGF-D combine with VEGFR-3 to regulate the survival and function of lymphatic endothelial cells [34]. NP-1 and NP-2 mainly exist in nerve axons and vascular endothelial cells. They can regulate the signal transduction of VEGFR-2 [35]. They are specific receptors of VEGF165.

At present, it has been recognized that the effect of VEGF on the proliferation and differentiation of endothelial cells is mediated by VEGFR-2. VEGFR-2 regulates the survival of endothelial cells. VEGF-A depends on VEGFR-2 and its subsequently activated PI3K (phosphatidylinositol 3-kinase) to regulate the survival of human umbilical vein endothelial cells, which is conducive to angiogenesis [31,32]. Second, VEGFR-2 regulates the migration of endothelial cells. The migration of endothelial cells is of great significance to angiogenesis. Endothelial cells can migrate along the concentration gradient of VEGF and other growth factors through the basement membrane hydrolyzed by protease, which plays a key role in metastasis [31,32]. Third, VEGFR-2 regulates the proliferation of endothelial cells. VEGF-A is the VEGF level of many endothelial cells. VEGFR-2 on the cell membrane combines with it to transmit extracellular signals to cells and activate a series of downstream signal pathways to regulate the proliferation of endothelial cells [31,32]. Fourth, VEGFR-2 regulates the permeability of endothelial cells. After VEGFR-2 is activated by VEGF binding, it stimulates vascular endothelial cells to release nitric oxide and causes changes in vascular permeability. The above effects are helpful to promote the survival, proliferation, and migration of endothelial cells and induce neovascularization [31,32].

Besides *VEGFR-2*, a previous genome-wide study revealed that the rs918519 SNP in *LOC285626*, rs1158314 in *NRXN3*, rs8004963 in *NRXN3*, rs11159428 in *NRXN3*, and rs918520 in *LOC285626* are associated with PDR [36]. Another study revealed that the -634G>C SNP in *VEGF* is associated with PDR [19]. Churchill et al. [17] showed that the -160C, rs13207351, and rs1570360 SNPs in *VEGF* are associated with PDR. So far, no SNPs in *VEGFR-2* have been associated with PDR. A previous study showed that the *VEGFR-2* rs2071559 SNP was associated with DR [23], but that previous study did not examine the association of the rs2071559 SNP with PDR. The present study examined the rs2071559 SNP with PDR, and it was not associated with DR in all subjects or with PDR among patients with DR. DR is a complex trait affected by the clinical characteristics of the T2DM patient, genetic background, and living environment [30]. The impact of the rs2071559 on DR can be affected by various other SNPs in the VEGF pathway, as well as in other pathways interacting with the VEGF pathway since DR is a polygenic disorder [29,30]. The relationship between the rs2071559 SNP and the development of PDR in Chinese may be weakened by the influence of other polymorphisms found in the Chinese population, but it will have to be examined. A Spanish study reported no association between the rs2071559 SNP and age-related macular degeneration [37]. Nevertheless, this SNP has been associated with various conditions, including age-related macular degeneration, stroke, endometriosis, hepatocellular carcinoma, and recurrent spontaneous abortion [15,16,20,38–40], but not with lymphoma [41]. In patients with T2DM, the rs2071559 SNP is associated with the extent of carotid atherosclerosis and with myocardial infarction [42,43].

Anti-VEGF drugs have become the first-line treatment for many retinal neovascularization diseases, including age-related macular degeneration and PDR [44,45]. Anti-VEGFR-2 drugs, including the monoclonal antibody ramucirumab and a variety of small-molecule tyrosine kinase inhibitors, remain solely used for the systemic treatment of malignant tumors (e.g., gastric and gastroesophageal adenocarcinoma in the second-line setting) [46], and are only in early trials for eventual ophthalmologic applications [47,48]. Therefore, in the context of future applications of VEGFR-2-targeting drugs in ophthalmic diseases, understanding the factors related to VEGFR-2 that can influence the course of the diseases is of importance. Future studies should examine all SNPs in *VEGFR-2* and the various proteins involved in its pathway.

Indeed, reliable SNPs could serve as predictive markers to make informed decisions about the management of specific diseases, the effectiveness of various drugs, and adverse reactions to specific drugs. The plasma sVEGFR-2 levels are elevated in severe DR, while plasma levels of sVEGFR-1 and sVEGFR-3 remain unaltered between patients and controls [49]. In this study, there were no associations between the *VEGFR-2* rs2071559 SNP and DR of PDR. VEGFR-2 controls endothelial cell proliferation, migration, survival, and osmosis via its activation by VEGF [8]. VEGFR-2 expression is higher in diabetic eyes than in controls [50]. The activation of VEGFR-2 by VEGF leads to nitric oxide production, blood vessel permeability, and endothelial proliferation [50]. An animal study showed that the specific knockdown of VEGFR-2 in the retina led to decreased neovascularization and improved the morphology of the endothelial cells, highlighting the central role of VEGFR-2 in retinal diseases [51]. Hence, in the context of future anti-VEGFR-2 therapies, a better understanding of the potential effects of VEGFR-2 in DR is essential.

This study has limitations. The sample size was small and from a single center. In addition, only one SNP was analyzed in this study because it was not studied before. Other *VEGFR-2* SNPs might yield different results.

This study showed no associations between the rs2071559 SNP and DR (among T2DM patients) or PDR (among DR patients). In the Han population in the Shijiazhuang area of China, the rs2071559 SNP of *VEGFR-2* is not associated with the risk of developing DR or PDR.

## Appendix

**Table A1:** Baseline characteristics of the subjects

	Controls *N* = 114	DM *N* = 123	NPDR *N* = 105	PDR *N* = 96
Age, years, median (range)	56.5 (43, 76)	61 (38, 82)^a^	62 (31, 81)^a^	60.5 (38, 79)
Male, *n* (%)	61 (53.5%)	56 (45.5%)	53 (50.5%)	35 (36.5%)
BMI, kg/m^2^, median (range)	24.5 (16.7, 35.8)	24.8 (16.8, 34.9)	25.4 (17.3, 48.9)	23.9 (18.3, 30.7)^c^
Systolic pressure, mmHg, median (range)	129.5 (95, 213)	128 (96, 180)	130 (80, 200)	140 (100, 200)^a,b^
Diastolic pressure, mmHg, median (range)	80 (40, 132)	80 (60, 100)	80 (50, 120)	80 (60, 110)^b^
Duration of DM, years, median (range)	—	7 (0.5, 24)	8 (1, 30)	10 (0, 28)^b,c^
Fasting blood glucose, mmol/L, median (range)	—	7 (4, 24)	7.5 (4.1, 19)	8 (3.52, 24)^b^

DM, diabetes mellitus without DR group; NPDR, non-proliferative DR group; PDR, proliferative DR group; BMI, body weight index.

Bonferroni *post hoc* test.

^a^Compared with control, *P* < 0.05.

^b^Compared with DM, *P* < 0.05.

^c^Compared with NPDR, *P* < 0.05.

**Table A2:** Hardy–Weinberg equilibrium

Group	*X* ^2^	*P*
PDR	0.98	0.32
NPDR	3.54	0.06
DM	0.72	0.40
Control	0.31	0.58

DM, diabetes mellitus without DR group; NPDR, non-proliferative DR group; PDR, proliferative DR group.
